# Complete genome sequence of biocontrol *Bacillus velezensis* strain F68 isolated from tomato rhizosphere soil

**DOI:** 10.1128/mra.00658-25

**Published:** 2026-02-11

**Authors:** Xiaomeng Liu, Zhenhe Su, Lihong Dong, Mingyuan Li, Shezeng Li, Qinggang Guo, Ping Ma

**Affiliations:** 1 Plant Protection Institute, Hebei Academy of Agriculture and Forestry Sciences, IPM Innovation Center of Hebei Province, International Science and Technology Joint Research Center on IPM of Hebei Province 91600, Baoding, China; 2 Hebei Agricultural Technology Extension Station (Hebei Fertilizer and Agricultural Water Saving Technology Center), Shijiazhuang, China; University of Strathclyde, Glasgow, United Kingdom

**Keywords:** genome sequence, *Bacillus velezensis*

## Abstract

*Bacillus* species are recognized as effective biological agents for inhibiting pathogenic microorganisms and promoting plant growth in agricultural application*s*. We report the complete genome sequence of *Bacillus velezensis strain* F68, which was isolated from tomato rhizosphere soil. The genome contains 3,972,640 bp of DNA with a GC content of 46.48%.

## ANNOUNCEMENT

*Bacillus velezensis* is recognized as a biocontrol agent with broad-spectrum antimicrobial activity (1). Strain F68 was isolated by us in 2021 from Shijiazhuang tomato rhizosphere soil (20 cm depth). Axenic culture was obtained by suspending 1 g of soil in 100 mL sterile water, heat-treating at 80°C for 10 min, serially diluting, plating on LB agar with 20 μg/mL fusaric acid (FA), incubating at 30°C for 24h, and three-zone streaking. The strain F68 achieved 83.33% control efficacy against tomato Fusarium wilt in greenhouse trials (2). Phylogenetic analysis of *gyrB* (OP717076), *rpoB* (OP717079), and *rpoC* (OP717082) genes confirmed its taxonomic classification as *B. velezensis* (2), and the strain has been deposited in CGMCC (No. 24598).

For DNA extraction, strain F68 was revived from −80°C glycerol stock (20%) on LB agar and grown overnight at 30°C. A single colony was inoculated into 200 mL of LB broth (1:100) and incubated at 30°C with shaking at 180 rpm for 12 h. Cells were harvested by centrifugation at 8,000 rpm for 10 min, and genomic DNA was extracted using the Wizard Genomic DNA Purification Kit (Promega, USA). DNA was quantified with a TBS-380 Fluorometer (Turner BioSystems Inc., Sunnyvale, CA, USA).

For Illumina sequencing, genomic DNA was fragmented into approximately 400 bp fragments using the Covaris M220 Focused Acoustic Shearer. Libraries were constructed with the NEXTflex Rapid DNA-Seq Kit 2.0 (Revvity, USA) and sequenced on an Illumina NovaSeq (Illumina, USA) sequencing platform in paired-end mode (2 × 150 bp), yielding 8,075,576 reads and 121,941,197 bp sequenced DNA. For Nanopore sequencing, genomic DNA was fragmented into 10 kb fragments using G-tubes (Covaris, MA, USA). Fragments were selected using magnetic beads, repaired using the NEBNext FFPE DNA Repair Mix and the NEBNext Ultra II End Repair/dA-Tailing Module (New England Biolabs), and processed with the EXP-NBD104/114 Native Barcoding Kit (Oxford Nanopore Technologies, UK) and SQK-LSK109 Ligation Sequencing Kit (Oxford Nanopore Technologies). The libraries were sequenced using an R9.4.1 MinION flow cell (FLO-MIN106) on a PromethION device. The 213,995 raw reads were basecalled and filtered using Guppy (V4.4.3) (3) in high-accuracy (hac) mode, producing 199,504 reads and 13,565,06,360 bp sequenced DNA. The average coverage depth was 341×, with the *N*_50_ of 3,964,303 bp.

Raw Illumina reads were quality-filtered and adapter-trimmed using fastp (V0.23.0) (4) with a minimum Phred score of 20 and a minimum length of 30 bp. Nanopore reads were processed with Guppy for real-time adapter trimming and filtering (*Q* ≥ 7, length ≥ 1,600 bp). The resulting clean reads (Illumina short-reads and ONT long-reads) were assembled using a hybrid approach with Unicycler (V0.4.8) (5), which generated two circular contigs (one chromosome, one plasmid). The assembly was polished with Pilon (V1.22) (6) using the Illumina reads, and the final sequence was annotated using the NCBI Prokaryotic Genome Annotation Pipeline (PGAP) (v6.10) (7). Unless otherwise noted, default parameters were used for all software.

The genome of strain F68 is 3,972,640 bp in total length with GC content of 46.48%, consisting of one circular double-stranded chromosome (3,964,303 bp) and one circular plasmid (8,337 bp), whose identity was confirmed using Plasmer (V0.1) (8). The annotated genome displays 3,936 genes, representing 3,741 protein-coding sequences, 86 transfer RNA genes, and 27 ribosomal RNA genes.

## Data Availability

The genome sequence of *Bacillus velezensis* strain F68 is available in GenBank under accession CP183293–CP183294. The raw Illumina and Oxford Nanopore Technologies (ONT) sequencing data for *B. velezensis* strain F68 have been deposited in the NCBI Sequence Read Archive (SRA) under accession numbers SRR34067083 and SRR35933124.
